# Diet–Microbiome–Brain Axis and Mental Health: Biological Mechanisms and Nutritional Implications

**DOI:** 10.3390/nu18091412

**Published:** 2026-04-29

**Authors:** Diana Uțu, Aniela-Roxana Nodiți-Cuc, Andreea-Mihaela Kiș, Ramona Amina Popovici, Dana Emanuela Pitic, Laria-Maria Trusculescu, Diana Marian, Andreea Georgiana Nan, Asad Salehi Matin, Dora Mihaela Cîmpian, Cristina Raluca Bodo, Alexandra Enache, Iustin Olariu

**Affiliations:** 1 Department II, Physiology and Pathophysiology, Faculty of Pharmacy, Victor Babeș University of Medicine and Pharmacy Timișoara, 2 Eftimie Murgu Square, 300041 Timișoara, Romania; diana.utu@umft.ro; 2 Department of Surgical Oncology, ‘Carol Davila’ University of Medicine and Pharmacy, 020021 Bucharest, Romania; aniela.noditi@umfcd.ro; 3 Department I, Management and Communication in Dental Medicine, Faculty of Dental Medicine, Victor Babeș University of Medicine and Pharmacy of Timișoara E. Murgu Square, Nr. 2, 300041 Timișoara, Romania; laria.trusculescu@umft.ro; 4 Doctoral School, Victor Babeș University of Medicine and Pharmacy Timișoara E. Murgu Square, Nr. 2, 300041 Timișoara, Romania; dana.pitic@umft.ro (D.E.P.); matin.asad-salehi@umft.ro (A.S.M.); 5 Department of Dentistry, Faculty of Dentistry, “Vasile Goldiş” Western University of Arad, 94-96 Revoluţiei Blvd., 310025 Arad, Romania; marian.diana@uvvg.ro (D.M.); olariu.iustin@uvvg.ro (I.O.); 6 First Clinic of Psychiatry, Clinical County Hospital of Targu Mures, 540142 Târgu Mureș, Romania; nan-dree96@yahoo.com; 7 Department of Bioethics, Medical Deontology and Medical Communication, “George Emil Palade” University of Medicine, Pharmacy, Science and Technology of Târgu Mureș, 540139 Târgu Mureș, Romania; do-ra.cimpian@umfst.ro (D.M.C.); cristina.bodo@umfst.ro (C.R.B.); 8 Department VIII, Discipline of Forensic Medicine, Bioethics, Deontology and Medical Law, Victor Babeș University of Medicine and Pharmacy Timișoara, E. Murgu Square, Nr. 2, 300041 Timișoara, Romania; enache.alexandra@umft.ro

**Keywords:** diet–microbiome–brain axis, gut microbiota, mental health, depression, anxiety, inflammation, psychobiotics, nutritional psychiatry

## Abstract

Background/Objectives: Diet is a primary and modifiable determinant of gut microbiota composition, diversity, and metabolic activity, thereby shaping microbial-derived metabolites, immune and inflammatory signalling, neuroendocrine regulation, and neural communication with the central nervous system. Western dietary patterns, characterised by high intake of ultra-processed foods, saturated fats, and low dietary fibre, are consistently associated with gut dysbiosis, impaired intestinal barrier function, chronic low-grade inflammation, and increased risk of depression, anxiety, cognitive impairment, and neurodegenerative disorders. Methods: This narrative review synthesises evidence from human observational studies, randomised controlled trials, animal models, and mechanistic investigations examining interactions among diet, gut microbiota, and mental health or neurobiological outcomes. Literature searches were conducted in PubMed, Scopus, and Web of Science for articles published up to December 2025. Results: The study highlights the therapeutic potential and limitations of dietary interventions, prebiotics, probiotics, and psychobiotics, and critically evaluates them. Also facilitates an improved understanding of diet–microbiome–brain interactions, which may help the development of personalised, nutrition-based strategies integrated into mental health prevention and clinical care. Conclusions: These findings support diet-based, microbiome-informed strategies as scalable adjuncts in mental health prevention and care.

## 1. Introduction

Mental health disorders, including depression and anxiety, constitute a major global public health burden, contributing substantially to disability, reduced quality of life, and increased healthcare costs worldwide [1,2]. Although pharmacological and psychotherapeutic interventions remain central to clinical management, treatment response is heterogeneous, and a substantial proportion of individuals experience partial or inadequate symptom relief [3,4]. These limitations have intensified interest in modifiable lifestyle factors that influence mental health trajectories, among which diet has emerged as a key determinant [1,5,6]. Diet influences brain health through both direct and indirect mechanisms. Beyond providing essential nutrients for neuronal structure and function, dietary patterns shape systemic metabolic and inflammatory states and profoundly affect the gut microbiome [7,8,9]. The gut microbiota is a complex and dynamic microbial ecosystem that regulates host metabolism, immune function, endocrine signalling, and neural communication [10,11]. Bidirectional interactions between the gut microbiota and the brain—collectively termed the gut–brain axis—operate through neural, immune, endocrine, and metabolic pathways, integrating peripheral physiological signals with central nervous system function [3,11,12]. Recent advances have identified diet as a primary upstream regulator of the gut–brain axis, giving rise to the concept of the diet–microbiome–brain axis, which integrates dietary exposures, microbial ecology, and neurobiological outcomes [5,6,13]. Dysregulation of this axis has been increasingly implicated in mood disorders, stress-related psychopathology, cognitive decline, and neurodegenerative disease [4,7,14,15,16]. Accumulating evidence suggests that diet-induced alterations in gut microbiota composition and metabolic output contribute to changes in intestinal permeability, immune activation, hypothalamic–pituitary–adrenal axis regulation, vagal nerve signalling, and neurotransmitter metabolism—mechanisms directly relevant to mental health [7,17,18,19,20,21,22,23]. In parallel, growing interest in nutritional psychiatry and microbiome-targeted interventions has highlighted the therapeutic potential of dietary modification, prebiotics, probiotics, and psychobiotics as adjunctive strategies for mental health prevention and treatment [5,6,10,24]. However, findings across studies remain heterogeneous due to variability in dietary exposures, microbial responsiveness, study design, and clinical populations, underscoring the need for integrative syntheses that critically evaluate biological mechanisms alongside clinical evidence [5,6,24].

Accordingly, this narrative review provides a comprehensive, mechanistically oriented synthesis of current evidence linking diet, the gut microbiota, and mental health outcomes. By integrating data from human observational studies, randomised controlled trials, animal models, and mechanistic research, this review aims to clarify key biological pathways underlying diet–microbiome–brain interactions and to inform future nutrition-based strategies for mental health promotion and clinical care. Dietary patterns have been associated not only with systemic health outcomes but also with broader developmental and inflammatory processes, underscoring the complex, multi-system interactions through which nutrition influences human health [25,26].

## 2. Materials and Methods

This narrative review synthesises evidence from human observational studies, randomised controlled trials, animal models, and mechanistic investigations examining interactions among diet, gut microbiota, and mental health or neurobiological outcomes. Literature searches were conducted in PubMed, Scopus, and Web of Science for articles published up to December 2025.

Search strategies included combinations of keywords and Medical Subject Headings (MeSH) such as “gut–brain axis”, “diet microbiome mental health”, “depression”, “anxiety”, “stress”, “psychobiotics”, “probiotics”, “prebiotics”, “dietary fiber”, “polyphenols”, “short-chain fatty acids”, and “hypothalamic–pituitary–adrenal axis”. Reference lists of relevant articles were also screened to identify additional pertinent studies. The study selection process was guided by predefined inclusion and exclusion criteria.

Only peer-reviewed articles published in English were considered. Studies were included if they examined dietary exposures or interventions in relation to gut microbiota composition or function and reported mental health, neuroendocrine, immune, or neurobiological outcomes. Dietary exposures were broadly defined to include both overall dietary patterns (e.g., Western diet, Mediterranean diet) and individual nutrients or components (e.g., dietary fibre, polyphenols, omega-3 fatty acids, probiotics, and prebiotics), in order to capture the complexity of diet–microbiome interactions.

Mental health outcomes encompassed both clinically diagnosed conditions (e.g., major depressive disorder, anxiety disorders) and validated self-reported measures, including standardised psychometric scales assessing depressive and anxiety symptoms, stress, and cognitive function.

Exclusion criteria included studies with insufficient methodological quality, very small sample sizes, unclear dietary exposure, absence of relevant mental health or neurobiological outcomes, non-peer-reviewed publications, and studies not available in English.

Given the narrative nature of the review, evidence was synthesised qualitatively, with particular emphasis on biological mechanisms linking diet-induced alterations in the microbiome to brain function and mental health. Priority was given to well-designed human studies and mechanistic experimental evidence where available.

## 3. Results

### 3.1. Gut Microbiota: Composition, Functional Roles, and Brain Health

#### Gut Microbiota: Taxonomic Composition and Determinants

The adult human gut microbiota is predominantly composed of bacteria belonging to the Firmicutes and Bacteroidetes phyla, with additional contributions from Actinobacteria, Proteobacteria, and Verrucomicrobia [5,10,27]. These phyla include key genera such as Bacteroides, Faecalibacterium, Bifidobacterium, Clostridium-related clusters, and Akkermansia, which collectively contribute to host metabolic, immune, and neuroactive functions [5,10,15]. Importantly, functional roles often differ even within the same taxonomic groups due to strain-level variation in metabolic capacity and gene expression, emphasising the need to complement taxonomic profiling with functional and metabolomic approaches [4,17].

Despite substantial interindividual variability in microbial composition, the gut microbiota exhibits pronounced functional redundancy, thereby preserving essential biological processes, such as complex carbohydrate fermentation, vitamin synthesis, and bile acid metabolism [5,7,27]. These conserved functional traits appear more stable than taxonomic composition and are central to host homeostasis [3].

Gut microbiota development begins at birth and is strongly influenced by early-life factors, including delivery mode, infant feeding practices, antibiotic exposure, diet, physical activity, and psychosocial stress. These determinants shape immune system maturation and neurodevelopmental trajectories, with perturbations during infancy associated with increased susceptibility to metabolic, autoimmune, and neuropsychiatric disorders later in life [8,11,28].

Microbial communities progressively stabilise throughout early childhood, reaching a relatively resilient adult-like configuration within the first years of life. While environmental and lifestyle factors remain the dominant modulators of microbiota composition across the lifespan, host genetics contributes modestly to the abundance of specific taxa involved in immune and metabolic regulation [10,28].

### 3.2. Core Functional Roles of the Gut Microbiota

#### 3.2.1. Fermentation of Dietary Substrates and Metabolite Production

Gut microorganisms ferment indigestible dietary fibres and resistant starches, leading to the production of short-chain fatty acids (SCFAs)—primarily acetate, propionate, and butyrate—which exert pleiotropic effects on host physiology [3,8,27]. SCFAs:

- Enhance intestinal epithelial barrier integrity;
- Regulate local and systemic immune responses;
- Influence lipid and glucose metabolism;
- Act as signalling molecules affecting brain function and neuroinflammatory processes [7,8,27].

Among SCFAs, butyrate plays a central role in neuroimmune regulation by promoting microglial maturation, inhibiting histone deacetylases, and modulating gene expression related to synaptic plasticity and neuroinflammation [7,13,27]. Reduced abundance of butyrate-producing bacteria has been associated with depressive symptoms, cognitive frailty, and neurodegenerative conditions [1,14,16].

#### 3.2.2. Modulation of Neuroactive Compounds and Neurotransmitters

The gut microbiota critically regulates tryptophan metabolism, a key biochemical pathway linking intestinal microbial activity to brain function [4,17,18]. Dietary tryptophan can be metabolised into several neuroactive compounds, including:

- Serotonin (5-hydroxytryptamine, 5-HT) modulates mood, appetite, sleep, pain perception, and gastrointestinal motility. Approximately 90% of systemic serotonin is synthesised in the gastrointestinal tract by enterochromaffin cells, with microbial signals influencing both serotonin biosynthesis and tryptophan availability [4,17,21].
- Kynurenine pathway metabolites, which play immunomodulatory and neuroactive roles. Dysregulation of this pathway has been implicated in depression, anxiety, cognitive impairment, ischemic stroke, and neuroinflammatory processes [18,19,29].
- Tryptamine and indole derivatives, which act as signalling molecules within the gut–brain axis, influence intestinal permeability, immune responses, and neuronal signalling [17,18].

Beyond tryptophan metabolism, gut microbes influence the synthesis and signalling of other neurotransmitters, including gamma-aminobutyric acid (GABA), dopamine, and glutamate, which are essential for inhibitory and excitatory neurotransmission, motivation, reward processing, and learning [15,20]. Alterations in these microbial–neurotransmitter interactions have been linked to mood disorders, anxiety, depression, and neurodevelopmental conditions [15,20].

#### 3.2.3. Immune System Regulation and Neuroimmune Crosstalk

The gut microbiota is tightly integrated with both the innate and adaptive immune systems, regulating immune cell differentiation, cytokine production, and systemic inflammatory tone [7,12,28]. Microbial metabolites and structural components interact continuously with host immune receptors, shaping immune tolerance and inflammatory responses.

**Figure 1:**
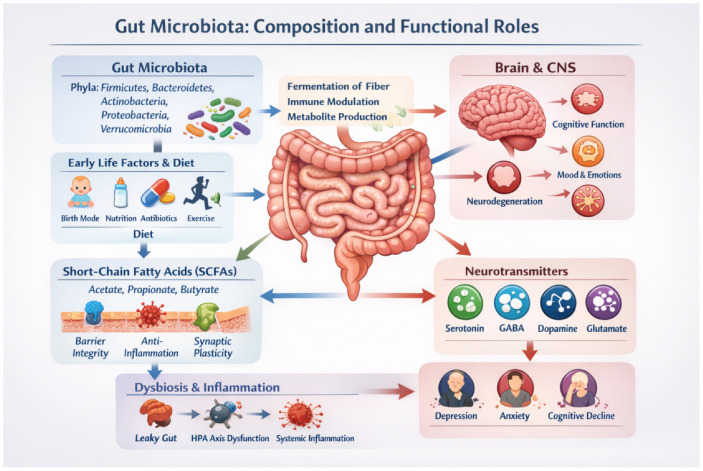
Gut microbiota composition and functional roles in the gut–brain axis: the adult human gut microbiota is dominated by bacteria from the Firmicutes and Bacteroidetes phyla, with additional contributions from Actinobacteria, Proteobacteria, and Verrucomicrobia. Gut microorganisms produce bioactive metabolites, including short-chain fatty acids (SCFAs) and neurotransmitters, which regulate intestinal barrier integrity, immune signalling, microglial maturation, and neuroinflammatory processes. Through metabolic, immune, endocrine, and neural pathways, including modulation of the hypothalamic–pituitary–adrenal axis and vagal nerve signalling, the gut microbiota communicates bidirectionally with the central nervous system, influencing stress responsivity, mood regulation, cognitive function, and mental health outcomes. **Abbreviations:** CNS, central nervous system; SCFAs, short-chain fatty acids; HPA, hypothalamic–pituitary–adrenal (axis); GABA, gamma-aminobutyric acid (the image was generated by IOpenAI, GPT-5.3).

Gut-derived immune signals modulate neuroimmune pathways, influencing microglial activation, blood–brain barrier permeability, and neural inflammation. Dysregulated microbiota–immune interactions have been implicated in the pathogenesis of multiple sclerosis, Alzheimer’s disease, Parkinson’s disease, depression, and anxiety disorders [7,16,21,27] (Figure 1).

#### 3.2.4. Gut–Brain Axis Mechanisms

The microbiota–gut–brain axis represents a complex bidirectional communication network involving:

- Neural pathways, particularly vagal afferents transmitting gut-derived signals to brain regions involved in emotion and cognition [12,21];
- Immune signalling, whereby cytokines and immune mediators influenced by microbial activity affect central nervous system function [7,16];
- Endocrine pathways, including modulation of the hypothalamic–pituitary–adrenal (HPA) axis and stress hormone secretion [13,21];
- Metabolic signalling, mediated by microbial metabolites such as SCFAs, bile acids, and tryptophan derivatives that engage receptors in peripheral tissues and the brain [4,7,27].

### 3.3. Microbiota and Brain Health: Evidence and Associations

#### 3.3.1. Mental Health and Behaviour

Observational human studies have consistently reported that gut microbiota dysbiosis is associated with depression and anxiety disorders. These associations involve altered serotonergic signalling, dysregulated tryptophan metabolism, immune activation, and HPA axis dysfunction [4,13,15,20,21].

#### 3.3.2. Neurodevelopment and Early-Life Programming

Early-life microbial colonisation plays a critical role in central nervous system development, influencing synaptic pruning, myelination, and neuroimmune maturation. Preclinical and translational studies indicate sensitive developmental windows during which microbiota perturbations exert long-lasting effects on behaviour, cognition, and stress responsiveness [12,28].

#### 3.3.3. Neurodegeneration and Ageing

Alterations in gut microbiota composition and microbial metabolites have been linked to Alzheimer’s and Parkinson’s diseases, through mechanisms involving chronic neuroinflammation, impaired gut barrier integrity, microglial dysfunction, and abnormal protein aggregation [1,7,16,27]. These interactions involve both immune-mediated and metabolite-mediated pathways and may contribute to disease onset and progression [1,14,16].

### 3.4. Dietary Modulation of the Gut Microbiome

Diet is one of the most influential and readily modifiable determinants of gut microbiota composition, diversity, and metabolic activity, exerting profound effects on host physiology and brain health. Both experimental and observational evidence demonstrate that short-term dietary interventions can rapidly reshape microbial community structure and metabolite profiles within days, whereas long-term dietary patterns promote the establishment of relatively stable and resilient microbial ecosystems that influence immune, metabolic, and neurobiological processes over time [5,8,28]. As such, diet represents a critical environmental factor through which lifestyle exposures modulate the microbiota–gut–brain axis.

Western dietary patterns, characterised by high intake of ultra-processed foods, saturated fats, and refined carbohydrates, are consistently associated with gut microbiota dysbiosis, including reduced microbial diversity, depletion of short-chain fatty acid (SCFA)-producing taxa, and enrichment of pro-inflammatory microorganisms [3,8,17]. Preclinical and mechanistic evidence indicate that SCFAs regulate intestinal barrier integrity, modulate immune responses, and influence microglial maturation [7,17]. Such inflammatory processes are mechanistically linked to dysregulation of immune signalling, activation of the hypothalamic–pituitary–adrenal (HPA) axis, and alterations in neurotransmitter metabolism, all of which play a central role in the development and persistence of depression and anxiety disorders [4,7,17]. Chronic inflammatory conditions characterised by complex biopsychosocial interactions have also been associated with adverse mental health outcomes and reduced quality of life [30]. Improving communication strategies in healthcare settings may contribute to better management of chronic conditions by supporting patient engagement, reducing anxiety, and enhancing therapeutic effectiveness [31].

A central mechanism linking diet, gut microbiota, and brain health involves the production of SCFAs, particularly acetate, propionate, and butyrate, which act as key mediators of microbiota–gut–brain communication. SCFAs regulate gut barrier function, modulate immune responses, influence microglial maturation, and affect neuroinflammatory and epigenetic pathways relevant to mood and cognition [22,32]. Reduced availability of SCFAs, commonly observed in Western dietary contexts, has been associated with increased neuroinflammation, HPA axis hyperactivity, and depressive-like behaviours [22,32].

In contrast, dietary patterns rich in dietary fibre, complex carbohydrates, polyphenols, omega-3 fatty acids, and fermented foods promote microbial diversity, functional redundancy, and metabolic resilience [5,8,25]. High-fibre diets selectively stimulate the growth of saccharolytic bacteria and enhance SCFA production, particularly butyrate, thereby supporting intestinal barrier integrity and exerting anti-inflammatory effects at both peripheral and central levels [22,33]. A recent dose–response meta-analysis demonstrated an inverse association between dietary fibre intake and the risk of depression, underscoring the relevance of fibre-driven modulation of the microbiota to mental health outcomes [33].

The Mediterranean diet, characterised by high consumption of fruits, vegetables, legumes, whole grains, nuts, olive oil, and fish, represents one of the most extensively studied dietary patterns in relation to gut microbiota and mental health. Adherence to Mediterranean-style diets has been shown to increase the abundance of beneficial taxa such as Faecalibacterium, Roseburia, and Bifidobacterium, enhance SCFA production, and reduce circulating inflammatory markers [7,8,34]. Randomised controlled trials and observational human studies suggest that adherence to a Mediterranean diet is associated with improved depressive symptoms [4,6,7,15,35]. Beyond macronutrient composition, dietary polyphenols play a critical role in shaping microbial metabolism and host neurobiology. Polyphenol-rich foods—including berries, cocoa, tea, coffee, and red wine—are extensively metabolised by gut microorganisms, generating bioactive metabolites with antioxidant, anti-inflammatory, and neuroactive properties [9,11,36]. These microbial-derived polyphenol metabolites have been shown to modulate neurotransmitter systems, reduce neuroinflammation, and support synaptic plasticity, suggesting protective effects against mood disorders and cognitive decline [9,36].

Dietary diversity itself has emerged as an additional determinant of microbiota richness and psychological resilience. Greater variety in dietary patterns has been associated with increased microbial diversity and lower depressive symptomatology, potentially through enhanced ecological stability and functional redundancy within the gut microbiome [37]. Conversely, nutrient inadequacy and monotonous dietary patterns may compromise microbiota-driven neuroprotective mechanisms, increasing vulnerability to mental health disorders [37].

Furthermore, fermented foods and probiotic-containing products may modulate the gut microbiota by introducing live microorganisms and fermentation-derived metabolites that influence microbial cross-feeding interactions and neuroactive compound production, including GABA and serotonin precursors [7,18]. Although individual responses vary, accumulating evidence supports the potential of fermented foods and probiotics as adjunctive strategies for improving mental well-being via microbiota-mediated pathways [20,27].

**Figure 2:**
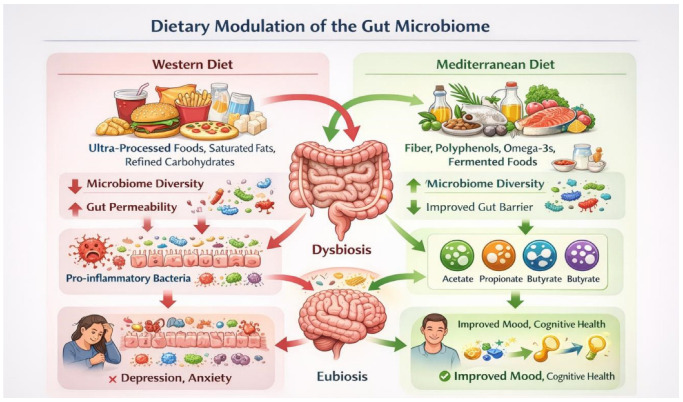
Dietary modulation of the gut microbiome and its implications for mental health. Western dietary patterns rich in ultra-processed foods, saturated fats, and refined carbohydrates are associated with reduced gut microbiota diversity, depletion of short-chain fatty acid (SCFA)–producing taxa, increased intestinal permeability, and chronic low-grade inflammation, thereby contributing to dysbiosis and increasing the risk of depression and anxiety. In contrast, Mediterranean-style dietary patterns characterised by high intake of dietary fibre, polyphenols, omega-3 fatty acids, and fermented foods promote microbial diversity, enhance SCFA production, improve gut barrier integrity, and support neuroprotective signalling along the gut–brain axis, ultimately contributing to improved mood and cognitive health. **Abbreviations:** SCFA, short chain fatty acid (the image was generated by OpenAI, GPT-5.3).

Collectively, these findings highlight diet as a central regulator of the gut microbiome and its downstream effects on immune, metabolic, and neurobiological processes. Dietary modulation of the gut microbiota represents a promising, low-risk, and scalable approach for the prevention and adjunctive management of depression, anxiety, and cognitive decline, warranting further investigation through well-controlled clinical trials and personalised nutrition strategies (Table 1) (Figure 2) [3,7,22,32].

### 3.5. Biological Mechanisms Linking Diet, Microbiome, and Brain

#### 3.5.1. Metabolic Pathways

Microbial metabolites represent a key mechanistic link between diet, gut microbiota, and brain function. Among these, short-chain fatty acids (SCFAs)—primarily acetate, propionate, and butyrate—play a central role in microbiota–gut–brain communication by modulating blood–brain barrier (BBB) integrity, microglial maturation, synaptic plasticity, and neuroinflammatory signalling. Preclinical evidence shows that the absence of the gut microbiota increases BBB permeability, whereas microbial recolonisation or SCFA supplementation restores tight-junction protein expression, providing direct evidence for microbiota-derived metabolic regulation of BBB function [36].

SCFAs are also essential for neuroimmune homeostasis, as studies in germ-free and antibiotic-treated mice demonstrate immature microglial phenotypes that can be partially reversed by SCFA administration [32]. Mechanistically, SCFAs act via G-protein–coupled receptors (e.g., FFAR2/FFAR3) and epigenetic pathways, including histone deacetylase (HDAC) inhibition, thereby influencing inflammatory gene expression and cellular metabolism [37,38].

At the clinical level, reduced SCFA production and depletion of butyrate-producing taxa, such as Faecalibacterium and Coprococcus, have been consistently reported in patients with major depressive disorder, with these alterations correlating with symptom severity [39]. Furthermore, altered faecal and circulating SCFA profiles are associated with depressive symptoms, gastrointestinal disturbances, and systemic inflammation, supporting their role in mood disorders [40].

Beyond SCFAs, microbial regulation of tryptophan metabolism represents another key pathway linking diet, microbiota, and brain function. Dietary tryptophan may be directed toward serotonin synthesis or diverted to the kynurenine pathway, with gut microbiota influencing this balance through substrate competition, immune modulation, and production of indole derivatives. Human dietary intervention studies further demonstrate that short-term nutritional changes can rapidly alter gut microbiota composition and circulating tryptophan metabolites [41]. Inflammation-induced activation of indoleamine 2,3-dioxygenase (IDO) shifts tryptophan metabolism toward the kynurenine pathway, leading to the accumulation of neuroactive metabolites such as quinolinic acid, which promotes excitotoxicity and neuroinflammation. Experimental models show that dysregulated kynurenine metabolism contributes to depressive-like behaviours and cognitive impairment, supporting a mechanistic link between microbiota-driven immune activation and altered neurotransmission [42].

Collectively, these findings indicate that diet-induced modulation of microbial metabolic outputs—particularly SCFAs and tryptophan-derived metabolites—directly influences BBB integrity, neuroimmune signalling, and synaptic function, thereby shaping emotional and cognitive outcomes. These pathways represent promising targets for microbiota-focused nutritional interventions aimed at preventing or ameliorating depression and other neuropsychiatric disorders, although well-controlled human trials integrating metabolomic and clinical endpoints remain urgently needed (Table 2) [36,37,38,39,40,41,42,43,44].

#### 3.5.2. Immune and Inflammatory Pathways

Diet-induced dysbiosis can increase intestinal permeability, facilitating the translocation of microbial components such as lipopolysaccharides (LPS) into the systemic circulation. This process, commonly referred to as metabolic endotoxemia, activates innate immune responses and promotes chronic low-grade inflammation, which has been implicated in the pathophysiology of depression, anxiety, and cognitive decline [44,45,46]. Elevated circulating LPS levels stimulate toll-like receptor 4 (TLR4) signalling on immune and endothelial cells, leading to the production of pro-inflammatory cytokines and chemokines that can influence central nervous system (CNS) function [39].

Pro-inflammatory cytokines—including interleukin-6 (IL-6), tumour necrosis factor-α (TNF-α), and interleukin-1β (IL-1β)—can access the brain through leaky regions of the blood–brain barrier or signal via afferent neural pathways, thereby directly interfering with neurotransmitter synthesis, synaptic plasticity, and neuroendocrine regulation [44,46]. Experimental studies demonstrate that peripheral immune activation reduces serotonin availability by inducing indoleamine 2,3-dioxygenase (IDO), diverting tryptophan metabolism toward the kynurenine pathway, and increasing the production of neurotoxic metabolites such as quinolinic acid [47]. These alterations contribute to excitotoxicity, impaired neurogenesis, and depressive-like behaviours in animal models [47,48].

Chronic low-grade inflammation also affects microglial activation states, shifting microglia toward a pro-inflammatory phenotype that exacerbates synaptic dysfunction and neuronal vulnerability. Animal studies show that high-fat or Western-style diets promote microglial activation in brain regions involved in mood regulation and cognition, including the hippocampus and prefrontal cortex, accompanied by increased cytokine expression and behavioural impairments [46,49]. Importantly, these neuroinflammatory changes can occur independently of overt obesity, highlighting the direct impact of diet-induced immune signalling on brain function [49].

In human studies, elevated inflammatory markers—such as C-reactive protein (CRP), IL-6, and TNF-α—have been consistently associated with depressive symptoms, cognitive impairment, and reduced treatment response to antidepressants [40,46]. Emerging evidence further suggests that individuals with increased intestinal permeability and altered gut microbiota composition exhibit higher systemic inflammatory burden and greater vulnerability to stress-related psychopathology [44,50].

Dietary modulation of the gut microbiota plays a critical role in regulating immune homeostasis. Diets rich in fermentable fibres and anti-inflammatory nutrients increase SCFA production, which exerts immunoregulatory effects by promoting regulatory T-cell differentiation, suppressing pro-inflammatory cytokine production, and enhancing gut barrier integrity [51]. In contrast, diets low in fibre and high in saturated fats favour pro-inflammatory microbial profiles and sustained immune activation [46,51].

Collectively, these findings support a mechanistic model in which diet-driven alterations in gut microbiota composition disrupt intestinal barrier function, activate innate immune pathways, and promote neuroinflammation, ultimately affecting neurotransmission, neuroplasticity, and behaviour. Targeting immune and inflammatory pathways through dietary and microbiota-focused interventions represents a promising strategy for mitigating depression, anxiety, and cognitive decline, although longitudinal human studies integrating immune, microbial, and neurobehavioural endpoints are still needed [44,45,46,47,48,49,50,51].

#### 3.5.3. Endocrine Pathways

The gut microbiota plays a critical role in regulating hypothalamic–pituitary–adrenal (HPA) axis activity, thereby shaping cortisol secretion, stress reactivity, and emotional behaviour. Pioneering studies in germ-free (GF) animals demonstrate that the absence of a commensal microbiota leads to exaggerated HPA axis responses to stress, characterised by elevated adrenocorticotropic hormone (ACTH) and corticosterone levels. Importantly, colonisation with specific microbial taxa or early-life microbial reconstitution can normalise these stress-induced endocrine responses, providing direct causal evidence for microbiota-dependent programming of the HPA axis [52].

Diet-induced dysbiosis has been shown to sensitise the HPA axis to both psychological and physiological stressors. Animal models exposed to high-fat or Western-style diets exhibit heightened corticosterone release and impaired negative feedback regulation of glucocorticoid signalling, effects that are accompanied by changes in gut microbial composition and increased intestinal permeability [53,54]. These endocrine alterations are associated with anxiety-like and depressive-like behaviours, suggesting that microbiota-driven endocrine dysregulation contributes to stress-related psychopathology [53].

Microbial metabolites serve as key mediators of microbiota–endocrine interactions. Short-chain fatty acids (SCFAs)can influence HPA axis activity indirectly by modulating immune signalling and directly by interacting with receptors expressed along the gut–brain axis. Experimental evidence indicates that SCFA supplementation attenuates stress-induced corticosterone secretion and reduces anxiety-like behaviours in rodents, effects that are partly mediated through vagal afferent signalling and modulation of hypothalamic neuropeptide expression [55]. In parallel, microbial regulation of tryptophan metabolism influences serotonin availability, which in turn modulates HPA axis feedback sensitivity and stress resilience [56].

The vagus nerve represents a major neuroendocrine conduit linking gut microbial activity to central stress circuits. Studies employing vagotomy or selective vagal blockade demonstrate that the anxiolytic and stress-attenuating effects of certain probiotics and dietary interventions are abolished in the absence of intact vagal signalling, underscoring the importance of gut–brain neural pathways in endocrine regulation [57]. These findings highlight the integrative nature of microbiota-mediated endocrine signalling, involving metabolic, neural, and immune components.

Human studies further support the relevance of microbiota–HPA axis interactions in mental health. Altered diurnal cortisol rhythms and elevated basal cortisol levels have been observed in individuals with depression and chronic stress, conditions frequently associated with gut microbiota dysbiosis and increased intestinal permeability [58]. Emerging clinical trials suggest that dietary interventions and probiotic supplementation can modulate cortisol responses to stress and improve stress-related symptoms, although interindividual variability and differences in microbial responsiveness remain significant challenges [59].

Mechanistic and experimental evidence suggest that gut microbiota influences tryptophan metabolism, while human studies provide emerging but still limited confirmation. Targeting microbiota–endocrine interactions through nutritional and microbial interventions represents a promising avenue for improving stress resilience and mental health, warranting further investigation in well-controlled longitudinal human studies [52,53,54,55,56,57,58,59].

#### 3.5.4. Neural Pathways

Neural communication between the gut and the brain occurs primarily via the vagus nerve, which serves as a rapid and bidirectional signalling conduit linking intestinal sensory inputs to central circuits involved in emotion, stress regulation, and cognition. Microbial metabolites, gut hormones, and immune mediators modulate vagal afferent activity either directly—through interaction with receptors expressed on vagal sensory neurons—or indirectly via activation of enteroendocrine and immune cells within the intestinal mucosa [60,61]. This neural pathway enables gut-derived signals to influence brain function on a timescale that is considerably faster than humoral or immune-mediated mechanisms.

Experimental evidence demonstrates that microbial metabolites, including short-chain fatty acids (SCFAs) and tryptophan-derived compounds, can alter vagal firing patterns and downstream neural activity in brain regions such as the nucleus tractus solitarius, amygdala, hippocampus, and prefrontal cortex—key nodes in emotional and cognitive processing [62]. Enteroendocrine cells act as critical intermediaries in this process by sensing luminal microbial products and releasing neuroactive peptides (e.g., cholecystokinin, glucagon-like peptide-1, peptide YY, and serotonin) that activate vagal afferents and modulate central neurotransmission [63].

Disruption of vagal signalling has been causally linked to altered emotional regulation and increased vulnerability to stress-related disorders. Preclinical studies employing subdiaphragmatic vagotomy or pharmacological vagal blockade show that the anxiolytic and antidepressant-like effects of specific probiotics, prebiotics, and dietary interventions are abolished in the absence of intact vagal pathways, underscoring the necessity of vagal integrity for microbiota-mediated behavioural effects [57,60]. These findings provide strong mechanistic evidence that vagal signalling is not merely correlative but functionally required for gut microbiota–driven modulation of mood and behaviour.

Neural plasticity within vagal circuits also appears sensitive to dietary and microbial influences. Chronic exposure to high-fat or Western-style diets alters vagal afferent sensitivity and reduces vagal tone, effects associated with impaired satiety signalling, heightened stress responsiveness, and cognitive deficits [64]. Conversely, diets rich in fermentable fibres and bioactive compounds enhance vagal signalling efficiency and promote adaptive neural responses, potentially through increased production of microbial metabolites and improved gut barrier function [62,64].

Human studies further support the relevance of gut–brain neural pathways in mental health. Reduced vagal tone, commonly assessed by heart rate variability (HRV), has been consistently associated with depression, anxiety, and cognitive impairment. Importantly, interventions that modify gut microbiota composition—such as dietary changes, probiotic supplementation, or consumption of fermented foods—have been shown to increase HRV and improve emotional regulation, suggesting enhanced vagal-mediated communication [65,66]. These observations align with emerging clinical interest in vagus nerve stimulation as a therapeutic strategy for treatment-resistant depression, reinforcing the clinical significance of gut–brain neural interactions [67].

**Figure 3:**
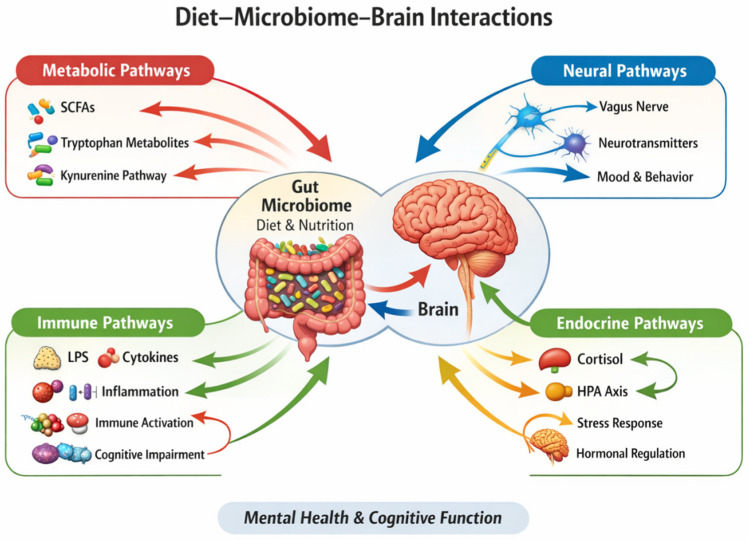
Schematic representation of the main biological pathways linking diet, gut microbiota, and brain function. **Abbreviations:** HPA, hypothalamic–pituitary–adrenal (axis); SCFAs, short-chain fatty acids; LPS, lipopolysaccharides.

Collectively, these findings indicate that diet- and microbiota-dependent modulation of vagal signalling constitutes a critical neural mechanism linking gut physiology to brain function. Through direct sensory signalling and interaction with enteroendocrine and immune pathways, the vagus nerve integrates microbial and dietary cues to regulate stress responses, emotional behaviour, and cognitive processes. Targeting gut–brain neural communication via nutritional and microbiota-focused interventions may therefore represent a promising avenue for enhancing mental health and stress resilience (Figure 3) [60,61,62,63,64,65,66,67].

### 3.6. Diet–Microbiome Interactions and Mental Health Outcomes

Large-scale human cohort studies and case–control investigations consistently report associations between gut microbiota composition and mental health phenotypes. Individuals with depression and anxiety show reduced abundance of butyrate-producing genera such as Faecalibacterium and Coprococcus, alongside increased pro-inflammatory taxa, with these microbial signatures correlating with symptom severity and quality-of-life scores [67,68]. Moreover, metagenomic analyses reveal that functional pathways related to SCFA biosynthesis and neurotransmitter metabolism are depleted in depressed individuals, suggesting that microbial function, rather than taxonomy alone, may be critical for mental health outcomes [69].

Dietary intake emerges as a major determinant of these microbiota–mental health associations. Prospective cohort studies demonstrate that higher intake of dietary fibre and plant-based foods is associated with reduced risk of depressive symptoms over time, partially mediated by favourable gut microbiota profiles and reduced systemic inflammation [2,70]. Conversely, high consumption of ultra-processed foods has been linked to increased depression risk and dysbiosis-associated inflammatory signatures [33].

Intervention studies further support a causal role for diet–microbiome modulation in mental health. Randomised controlled trials show that adherence to a Mediterranean-style diet significantly improves depressive symptoms compared with social support or habitual diet controls, with concurrent changes in gut microbiota composition and inflammatory markers [71]. Additionally, dietary interventions enriched in fermentable fibres and polyphenols have been shown to increase SCFA production and improve stress resilience and cognitive performance in both healthy adults and clinical populations [72,73].

Emerging metabolomic studies indicate that gut microbiota-derived metabolites, including SCFAs and indole derivatives, mediate these effects by influencing immune activation, HPA axis regulation, and neural signalling pathways [24,74]. Collectively, these findings strengthen the evidence that diet–microbiome interactions play a contributory role in the onset, progression, and potential treatment of mental health disorders.

#### 3.6.1. Depression and Anxiety Disorders

Beyond immune activation, dysbiosis-related alterations in microbial metabolic output contribute to disrupted neurotransmitter homeostasis in depressive and anxiety disorders. Reduced availability of microbiota-derived short-chain fatty acids (SCFAs), particularly butyrate, has been associated with impaired blood–brain barrier integrity, enhanced microglial activation, and increased neuroinflammatory tone, processes that may exacerbate mood dysregulation and anxiety-related behaviours [37,75]. Human studies further indicate that faecal and circulating SCFA profiles correlate with depressive symptom severity and inflammatory markers, supporting a role for impaired microbial metabolic support of central nervous system function in affective disorders [76].

Altered microbial metabolism of tryptophan represents an additional pathway linking dysbiosis to depression and anxiety. Increased inflammatory signalling promotes activation of indoleamine 2,3-dioxygenase (IDO), diverting tryptophan away from serotonin synthesis toward the kynurenine pathway. This shift results in the accumulation of neuroactive metabolites such as quinolinic acid, which can induce excitotoxicity, impair synaptic plasticity, and contribute to depressive-like behaviour [48]. Clinical evidence demonstrates altered kynurenine-to-tryptophan ratios in patients with major depressive disorder, further implicating microbiota–immune interactions in the pathophysiology of mood disorders [77].

Endocrine dysregulation also contributes to the gut–brain interactions observed in depression and anxiety. Dysbiosis and increased intestinal permeability are associated with altered hypothalamic–pituitary–adrenal (HPA) axis activity, including elevated basal cortisol levels and impaired negative feedback regulation. These endocrine abnormalities reinforce inflammatory signalling and neurotransmitter imbalance, thereby sustaining affective symptomatology [37,58]. Notably, restoration of microbial balance through dietary or probiotic interventions has been shown to attenuate stress-induced HPA axis activation in experimental models, suggesting a degree of reversibility in these pathways [75].

Dietary patterns rich in fermentable fibres, polyphenols, and other plant-derived bioactive compounds may counteract these mechanisms by enhancing SCFA production, strengthening gut barrier integrity, and reducing systemic inflammation. Observational and interventional studies consistently associate higher diet quality with lower prevalence and severity of depressive and anxiety symptoms, supporting the role of diet-driven microbiome modulation as a complementary strategy in affective disorder management [1,75,76].

#### 3.6.2. Stress Responsivity and the HPA Axis

Sustained activation of the hypothalamic–pituitary–adrenal (HPA) axis exerts profound effects on gut physiology, including alterations in epithelial barrier function, mucus secretion, and immune surveillance, all of which contribute to shifts in gut microbial composition. Elevated glucocorticoid levels have been shown to influence bacterial growth dynamics and virulence directly, favouring stress-associated microbial profiles and reducing commensal resilience [58]. These stress-induced microbial changes may persist beyond the acute stressor, suggesting that chronic HPA axis activation can induce long-lasting alterations in host–microbiome interactions.

Experimental models further demonstrate that psychological stress reduces the abundance of beneficial taxa and decreases microbial diversity, while increasing the relative abundance of opportunistic and pro-inflammatory microorganisms. These changes are accompanied by reductions in short-chain fatty acid (SCFA) production and increased intestinal permeability, reinforcing systemic inflammatory signalling and impairing negative feedback regulation of the HPA axis [78]. Such findings support the existence of a self-reinforcing stress–microbiome loop, wherein stress-induced dysbiosis perpetuates neuroendocrine dysregulation.

Microbial metabolites play a key role in modulating stress responsiveness. SCFAs have been shown to attenuate stress-induced corticosterone release and normalise HPA axis activity in animal models, potentially through interactions with vagal afferent pathways and hypothalamic glucocorticoid receptor signalling [79]. In contrast, dysbiosis-associated alterations in tryptophan metabolism may exacerbate stress sensitivity by reducing central serotonin availability and increasing activation of the kynurenine pathway, further amplifying HPA axis reactivity [80].

Human studies provide converging evidence for the involvement of the microbiota in stress responsivity. Individuals exposed to chronic psychosocial stress exhibit altered gut microbiota composition, increased intestinal permeability, and elevated inflammatory and cortisol levels, with these parameters correlating with perceived stress and anxiety scores [81].

Importantly, dietary quality appears to modulate these relationships: diets rich in fermentable fibres, polyphenols, and micronutrients are associated with reduced cortisol output and improved stress resilience, whereas Western dietary patterns exacerbate stress-related endocrine and microbial disturbances [82,83].

Together, these findings highlight the central role of diet in shaping the bidirectional interactions between stress, gut microbiota, and the HPA axis. By modulating microbial composition, metabolic output, and immune signalling, diet can either exacerbate or buffer stress-induced neuroendocrine dysregulation, underscoring its relevance as a modifiable factor in stress-related mental health disorders.

#### 3.6.3. Cognitive Function, Ageing, and Neurodegeneration

Accumulating evidence from both human and animal studies indicates that age-related alterations in gut microbiota composition contribute to cognitive decline and increased vulnerability to neurodegenerative disorders. Ageing is commonly associated with reduced microbial diversity, decreased abundance of butyrate-producing bacteria, and enrichment of pro-inflammatory taxa. These changes correlate with frailty, impaired cognitive performance, and elevated neuroinflammatory markers in older adults [82,84]. Age-related dietary changes, including reduced dietary fibre intake and increased consumption of ultra-processed foods, frequently exacerbate these microbial shifts.

Microbiota-derived short-chain fatty acids (SCFAs) play a central role in maintaining cognitive function and brain homeostasis during ageing. Experimental studies demonstrate that reduced SCFA availability compromises blood–brain barrier integrity, increases permeability to peripheral inflammatory mediators, and promotes microglial activation, thereby facilitating neuroinflammatory cascades that accelerate cognitive decline [85,86]. In parallel, SCFAs exert immunomodulatory effects by regulating regulatory T-cell homeostasis and suppressing pro-inflammatory immune responses, mechanisms that indirectly support neuronal function and synaptic integrity [87].

In the context of neurodegenerative diseases, distinct gut microbiota signatures have been identified in both Alzheimer’s and Parkinson’s disease. Human studies in Alzheimer’s disease report significant alterations in gut microbiota composition, accompanied by changes in inflammatory and metabolic profiles that correlate with cognitive impairment severity [88]. Similarly, preclinical models of Parkinson’s disease demonstrate that gut microbiota dysbiosis exacerbates motor deficits and neuroinflammation. At the same time, modulation of the microbial composition attenuates disease-related neuropathology, providing causal evidence for the microbiota’s involvement in neurodegenerative processes [89,90].

Metabolomic analyses further support a role for microbiota-derived metabolites in cognitive dysfunction and neurodegeneration. Altered profiles of SCFAs and other microbial metabolites have been observed in patients with Alzheimer’s disease and are associated with impaired synaptic function, increased neuroinflammation, and cognitive decline [91]. Collectively, these findings suggest that diet-driven alterations in gut microbiota composition and metabolic output may contribute to brain ageing and neurodegenerative disease progression through combined effects on barrier integrity, immune activation, and neuronal signalling.

Although causal relationships in humans remain incompletely defined, the convergence of observational, experimental, and mechanistic evidence supports a contributory role of diet–microbiome interactions in cognitive ageing and neurodegeneration, highlighting the gut microbiota as a potential target for nutritional and lifestyle-based interventions aimed at preserving cognitive health.

#### 3.6.4. Bidirectional Interactions Between Diet, Stress, and the Microbiome

Experimental evidence indicates that psychological stress can directly reshape gut microbial communities through stress hormone-mediated mechanisms. Glucocorticoids and catecholamines released during stress exposure alter gut motility, mucosal secretion, and immune surveillance, creating an intestinal environment that favours the expansion of stress-responsive and pro-inflammatory bacterial taxa [92,93,94]. These stress-induced microbial shifts are accompanied by reductions in short-chain fatty acid (SCFA) production and increased intestinal permeability, which further amplify systemic inflammation and neuroendocrine dysregulation.

Dietary composition strongly modulates the magnitude and persistence of stress-induced alterations in the microbiome. Animal studies demonstrate that exposure to chronic stress in the context of a high-fat or low-fibre diet results in more pronounced dysbiosis, exaggerated inflammatory responses, and heightened hypothalamic–pituitary–adrenal (HPA) axis activation compared with stress exposure under fibre-rich dietary conditions [95,96,97]. These findings suggest that diet quality determines whether stress-related microbiome changes remain transient or evolve into sustained dysregulation.

Conversely, diets enriched in fermentable fibres and plant-derived bioactive compounds appear to confer protection against stress-induced disturbances in the microbiome. Increased fibre intake enhances microbial production of SCFAs, which exert anti-inflammatory effects, support epithelial barrier integrity, and modulate vagal afferent signalling involved in stress regulation [96]. In experimental models, dietary fibre supplementation attenuates stress-induced corticosterone release and reduces anxiety-like behaviour, effects that are accompanied by preservation of microbial diversity and metabolic function.

Human studies further support the interactive role of diet and stress in shaping gut microbiota and mental health outcomes. Individuals experiencing high psychosocial stress exhibit more pronounced alterations in gut microbiota and inflammatory profiles when consuming low-quality diets. In contrast, higher diet quality is associated with attenuated cortisol responses and greater microbial stability under stress [97]. These observations reinforce the concept that diet acts as a context-dependent modifier of stress–microbiome–brain interactions rather than an isolated exposure.

Collectively, these findings highlight that the impact of psychological stress on gut microbiota and mental health is strongly conditioned by dietary context. Integrating dietary strategies that support microbial resilience with stress-reduction approaches may therefore represent an effective avenue for mitigating stress-related dysbiosis and its downstream neurobiological consequences.

### 3.7. Nutritional and Microbiome-Targeted Interventions

Dietary interventions aimed at restoring gut microbiota balance represent a promising strategy for the prevention and adjunctive treatment of mental health disorders [5,7,9]. Whole-food dietary patterns rich in dietary fibre, polyphenols, and omega-3 fatty acids are consistently associated with beneficial effects on microbial composition and metabolic function. Among these, Mediterranean-style dietary patterns demonstrate the most consistent associations with improved mental health outcomes, including reduced depressive and anxiety symptoms, alongside increased microbial diversity, enhanced SCFA production, and reduced inflammatory markers [7,9,14]. In addition to dietary patterns, microbiome-targeted approaches, such as probiotics, prebiotics, and psychobiotics, have shown modest yet significant benefits. However, heterogeneity across studies, including variability in microbial strains, dosages, and individual responses, highlights the need for more standardised and personalised approaches [5,10]. Notably, some studies also demonstrate normalisation of hypothalamic–pituitary–adrenal (HPA) axis activity and reduced cortisol responses to stress, supporting mechanistic links between microbial modulation and neuroendocrine regulation.

Randomised controlled trials and meta-analytic evidence suggest that prebiotic interventions may influence mental health outcomes, highlighting the importance of microbial metabolic activity. Supplementation with fermentable fibres, such as inulin-type fructans and galacto-oligosaccharides, selectively promotes the growth of beneficial gut bacteria and enhances SCFA production. Experimental and clinical studies suggest that these changes support gut barrier integrity, attenuate systemic inflammatory signalling, and improve stress resilience, with behavioural effects closely linked to microbial metabolite profiles rather than taxonomic shifts alone [5,22].

Despite these encouraging findings, substantial heterogeneity persists across dietary and microbiome-targeted intervention studies. Variability in microbial strains, dosages, intervention duration, baseline microbiome composition, and clinical populations complicates interpretation and limits generalisability. These limitations underscore the need for more standardised, mechanistically informed trial designs and for integrating microbiome and metabolomic profiling to identify responders and optimise intervention strategies. Personalised nutrition approaches that account for individual microbial and metabolic characteristics may enhance the precision and effectiveness of future mental health interventions.

### 3.8. Clinical Implications for Mental Health and Nutrition Practice

Growing evidence supports integrating nutrition-focused strategies into mental health prevention and care, with diet quality emerging as a modifiable, clinically actionable determinant of psychological well-being. Routine assessment of dietary patterns—alongside sleep, physical activity, and psychosocial stress—may provide valuable insight into modifiable contributors to depressive, anxiety, and stress-related symptoms.

From a practical standpoint, fibre-rich, Mediterranean-style dietary patterns represent a feasible and scalable intervention that can be implemented in both clinical and community settings. Emphasising whole grains, legumes, fruits, vegetables, nuts, olive oil, and fish may support mental health by enhancing short-chain fatty acid production, strengthening gut barrier integrity, and attenuating systemic inflammation. Importantly, these dietary recommendations align with existing cardiometabolic and public health guidelines, facilitating their translation into routine practice without additional clinical burden.

Microbiome-targeted interventions, including probiotics, prebiotics, and psychobiotics, may offer additional benefit as adjunctive strategies, particularly in individuals with stress-related disorders, low-grade inflammation, or suboptimal response to standard pharmacological or psychotherapeutic treatments. Current evidence supports cautious, strain-specific use of these interventions, with realistic expectations regarding their modest but potentially meaningful effects on mood, stress reactivity, and quality of life.

Interindividual variability in gut microbiota composition, dietary habits, metabolic status, and stress exposure underscores the importance of personalised and context-sensitive approaches. Rather than universal supplementation, nutrition-based mental health interventions may be most effective when tailored to baseline dietary patterns, gastrointestinal symptoms, and lifestyle factors. Incorporating simple dietary screening tools and, where feasible, microbiome-informed insights may enhance clinical decision-making and responsiveness to interventions.

Finally, optimal mental health outcomes are unlikely to result from dietary modification in isolation. Integrating nutrition strategies with broader lifestyle interventions, including stress management, sleep optimisation, and physical activity, may maximise therapeutic benefit by simultaneously targeting multiple components of the diet–microbiome–brain axis. Such integrative approaches are particularly well-suited to preventive care and early intervention, offering low-risk, accessible, and sustainable options to support long-term mental health. Behavioural determinants of health prevention, including risk perception and adherence to preventive strategies among healthcare students, may also influence lifestyle-related exposures such as diet quality and stress management [98].

## 4. Limitations and Future Perspectives

Although substantial progress has been made in elucidating the links between diet, gut microbiota, and brain health, several important limitations should be acknowledged. First, a large proportion of the available human evidence remains observational, which constrains causal inference. While associations between dietary patterns, microbiota composition, and mental health outcomes are robust and consistent, they cannot fully exclude residual confounding by lifestyle, socioeconomic, or genetic factors. Consequently, causality cannot be definitively established without well-controlled longitudinal and interventional studies.

Second, heterogeneity across dietary and microbiome-targeted intervention studies represents a major challenge. Differences in dietary assessment methods, probiotic or prebiotic strains, dosages, intervention duration, baseline microbiome composition, and clinical populations complicate comparisons across studies and limit generalisability. In addition, mental health outcomes are often assessed using diverse psychometric tools, further contributing to variability in reported effects.

Third, many studies rely primarily on taxonomic profiling of the gut microbiota, which provides limited insight into functional capacity. Emerging evidence suggests that microbial metabolic output—rather than microbial composition alone—may be more directly relevant to brain health. The lack of integrated multi-omics approaches (e.g., metagenomics, metabolomics, immune profiling) in many studies restricts mechanistic interpretation and hampers the identification of key causal pathways.

Future research should prioritise mechanistically informed, longitudinal, and randomised controlled trials that integrate detailed dietary assessment with comprehensive microbiome and metabolomic analyses, alongside standardised neuropsychological and psychiatric outcomes. Particular emphasis should be placed on identifying microbial and metabolic signatures predictive of treatment response, thereby enabling personalised nutrition and microbiome-based interventions.

Life-course approaches also warrant greater attention. Early life, adolescence, pregnancy, and older age represent periods of heightened microbiome and neurobiological plasticity during which dietary modulation may exert long-lasting effects on mental health trajectories. Finally, future studies should adopt integrative frameworks that consider diet in conjunction with psychological stress, sleep, physical activity, and other lifestyle factors, reflecting the multifactorial nature of mental health disorders.

While a substantial proportion of mechanistic evidence derives from preclinical models, human studies—particularly randomised controlled trials—provide increasing support for the clinical relevance of diet–microbiome–brain interactions, although causal pathways remain only partially established.

## 5. Clinical Takeaways

- Diet quality represents a modifiable factor with clinically relevant impact on mental health, acting through microbiome-mediated mechanisms.
- Dietary patterns rich in fibre, polyphenols, and omega-3 fatty acids support gut microbiota diversity, enhance short-chain fatty acid production, and may contribute to improved mood and cognitive function.
- Western dietary patterns are associated with dysbiosis, chronic low-grade inflammation, and increased risk of depression and anxiety.
- Microbiome-targeted interventions, including probiotics, prebiotics, and psychobiotics, may offer modest benefits as adjunctive strategies, particularly in stress-related and inflammatory conditions.
- Individual variability in microbiota composition highlights the importance of personalised and context-dependent nutritional approaches.
- Integration of dietary strategies with broader lifestyle interventions (e.g., stress management, sleep, physical activity) may enhance overall mental health outcomes.

## 6. Conclusions

This review contributes to the growing field of nutritional psychiatry by providing a structured, mechanism-based synthesis across multiple levels of evidence. In conclusion, converging evidence indicates that diet is a central and modifiable determinant of gut microbiota composition and function, with significant implications for brain health and mental well-being. Through interconnected metabolic, immune, endocrine, and neural pathways, the gut microbiome mediates key aspects of gut–brain communication, influencing stress responsivity, mood regulation, cognitive function, and vulnerability to neuropsychiatric and neurodegenerative disorders.

A key contribution of this review lies in its integrative, mechanism-oriented synthesis of evidence linking dietary exposures, microbial metabolic activity, and neurobiological outcomes across multiple levels of investigation. By explicitly bridging findings from human studies, preclinical models, and mechanistic research, this work provides a more comprehensive framework for understanding the diet–microbiome–brain axis.

From a clinical perspective, dietary patterns rich in fibre, plant-derived bioactive compounds, and minimally processed foods—particularly Mediterranean-style diets—offer feasible, scalable strategies that may support mental health through microbiome-mediated mechanisms. Microbiome-targeted interventions, including prebiotics, probiotics, and psychobiotics, may offer additional benefits as adjunctive approaches, although their effects remain modest and context-dependent.

Future research should prioritise well-designed longitudinal and randomised controlled trials integrating detailed dietary assessment with microbiome and metabolomic profiling, alongside standardised mental health outcomes. Greater emphasis on personalised nutrition approaches, responder identification, and multi-omics integration will be essential to translate current knowledge into clinical practice.

Overall, advancing the integration of nutritional science, microbiome research, and neuroscience holds significant potential for developing low-risk, accessible, and scalable strategies to improve mental health and cognitive resilience.

## Figures and Tables

**Table 1:** Effects of dietary patterns on gut microbiota composition and mental health outcomes.

Dietary Pattern/Component	Effects on Gut Microbiota	Key Mechanisms	Mental Health Outcomes
Western diet (ultra-processed foods, saturated fats)	↓ microbial diversity; ↓ SCFA-producing taxa; ↑ pro-inflammatory bacteria	Gut barrier dysfunction; metabolic endotoxemia; chronic inflammation	↑ risk of depression and anxiety
Dietary fibre (whole grains, legumes, vegetables)	↑ SCFA-producing bacteria (Faecalibacterium, Roseburia)	Enhanced gut barrier integrity; immune regulation	Improved mood; reduced depressive symptoms
Mediterranean diet Polyphenol-rich foods (fruits, vegetables, olive oil) Fermented foods (yoghurt, kefir, fermented vegetables)	↑ microbial diversity; ↑ Bifidobacterium; ↑ SCFAs	Anti-inflammatory signalling; neuroprotection	Lower prevalence and severity of depression and anxiety
	Modulation of microbial metabolism; ↑ beneficial taxa	Production of neuroactive and antioxidant metabolites	Cognitive and emotional resilience
	↑ functional microbial resilience	Neurotransmitter modulation; immune balance	Stress reduction; improved psychological well-being
Omega-3 fatty acids (fish, nuts, seeds) Dietary Pattern/Component	Indirect support of beneficial microbiota	Anti-inflammatory effects; membrane signalling	Protective effects against mood disorders
	Effects on Gut Microbiota	Key Mechanisms	Mental Health Outcomes
Western diet (ultra-processed foods, saturated fats) Dietary fibre (whole grains, legumes, vegetables) Mediterranean diet	↓ microbial diversity; ↓ SCFA-producing taxa; ↑ pro-inflammatory bacteria	Gut barrier dysfunction; metabolic endotoxemia; chronic inflammation	↑ risk of depression and anxiety
	↑ SCFA-producing bacteria (Faecalibacterium, Roseburia)	Enhanced gut barrier integrity; immune regulation	Improved mood; reduced depressive symptoms
	↑ microbial diversity; ↑ Bifidobacterium; ↑ SCFAs	Anti-inflammatory signalling; neuroprotection	Lower prevalence and severity of depression and anxiety
Polyphenol-rich foods (fruits, vegetables, olive oil) Fermented foods (yoghurt, kefir, fermented vegetables)	Modulation of microbial metabolism; ↑ beneficial taxa	Production of neuroactive and antioxidant metabolites	Cognitive and emotional resilience
	↑ functional microbial resilience	Neurotransmitter modulation; immune balance	Stress reduction; improved psychological well-being

**Table 2:** Biological mechanisms linking diet, gut microbiota, and brain function. **Abbreviations:** SCFAs, short-chain fatty acids; CNS, central nervous system; GABA, gamma-aminobutyric acid.

Microbial Component/Metabolite	Main Dietary Source	Biological Mechanism	Effects on Brain and CNS
Short-chain fatty acids (SCFAs) (acetate, propionate, butyrate) Butyrate	Fermentable dietary fibres	Microbial fermentation in the colon; regulation of blood–brain barrier permeability; modulation of microglial activation	Reduced neuroinflammation, enhanced synaptic plasticity and cognitive function; decreased levels reported in depression and neurodegenerative disorders
	Dietary fibre, resistant starch	Histone deacetylase inhibition: primary energy source for colonocytes	Neuroprotective effects: improves memory and reduces neuroinflammatory signalling
Tryptophan metabolism Kynurenine and downstream metabolites	Dietary proteins	Microbial conversion of tryptophan toward serotonin or kynurenine pathways	Modulates mood, stress responsiveness, and cognitive performance
	Tryptophan	Immune activation and inflammatory signalling; ability to cross the blood–brain barrier	Associated with depressive symptoms, anxiety, and cognitive decline
Microbial-derived neurotransmitters (GABA, serotonin, dopamine) Microbial component/metabolite	Fibre-rich and diverse diets	Direct or indirect synthesis by gut microbiota	Regulation of emotional behaviour, stress response, and neurocognitive processes
	Main dietary source	Biological mechanism	Effects on brain and CNS
Short-chain fatty acids (SCFAs) (acetate, propionate, butyrate)	Fermentable dietary fibres	Microbial fermentation in the colon; regulation of blood–brain barrier permeability; modulation of microglial activation	Reduced neuroinflammation, enhanced synaptic plasticity and cognitive function; decreased levels reported in depression and neurodegenerative disorders

## Data Availability

No new data were created or analysed in this study. Data sharing is not applicable to this article.

## Abbreviations

The following abbreviations are used in this manuscript:

ACTH	adrenocorticotropic hormone
BBB	Blood–brain barrier
CNS	Central nervous system
CRP	C-reactive protein
GABA	Gamma-aminobutyric acid
HDAC	Histone deacetylase
HPA	Hypothalamic–pituitary–adrenal
HRV	Heart rate variability
IDO	Indoleamine 2,3-dioxygenase
IL-6	Interleukin-6
IL-1β	Interleukin-1β
LPS	Lipopolysaccharides
MDD	Major depressive disorder
SCFAs	Short-chain fatty acids
TLR4	Toll-like receptor 4 TLR4
TNF-α	Tumour necrosis factor-α
